# *RORA* and Autism in The Isfahan Population: Is There An Epigenetic Relationship

**DOI:** 10.22074/cellj.2016.4720

**Published:** 2016-09-26

**Authors:** Mansoor Salehi, Elahe Kamali, Mojgan Karahmadi, Seyyed mohammad Mousavi

**Affiliations:** 1 Department of Genetics and Molecular Biology, School of Medicine, Isfahan University of Medical Sciences, Isfahan, Iran; 2 Division of Genetics, Department of Biology, Faculty of Science, University of Isfahan, Isfahan, Iran; 3 Behavioral Sciences Research Center, Isfahan University of Medical Sciences, Isfahan, Iran; 4 Genetic and Identification Lab, Legal Medicine Center, Isfahan, Iran; 5 Cellular and Molecular Research Center, School of Medicine, Shahrekord University of Medical Sciences, Shahrekord, Iran

**Keywords:** Autism, Epigenetics, Methylation, *RORA*, Promoter

## Abstract

**Objective:**

Autism is a neurodevelopmental disorder characterized by difficulty in verbal and non-verbal communication, impaired social interaction, and restricted and repetitive behavior. It has been recently introduced as a multigenic disorder with significant epigenetic effects on its pathology. Recently, epigenetic silencing of retinoic acid receptor- related orphan receptor alpha (*RORα*) gene (which has an essential role in neural tissue development) was shown to have occurred in autistic children due to methylation of its promoter region. This may thus explain a significant part of the molecular pathogenesis of autism. Therefore, we aimed to confirm this finding by implementing a case-control (experimental) study in the population of Isfahan.

**Materials and Methods:**

The methylation status of a 136 bp sequence of a GpG island (encompassing 13 CpG sites) in the *RORA* promoter region (positions -200 to -64) as an experimental study was examined in the lymphocyte cells of 30 autistic children after sodium bisulfite treatment using the melting curve analysis-methylation (MCA-Meth) assay compared with normal children. Also, quantitative reverse transcriptase-polymerase chain reaction (qRT-PCR) analysis was used to estimate the level of mRNA transcripts and to evaluate MCA-Meth analysis results.

**Results:**

This study revealed no methylation in the examined promoter regions in both autistic and normal children, with the melting curve of all studied samples being comparable to that of the non-methylated control. The results of MCA-Meth analysis were also consistent with qRT-PCR results. We therefore observed no significant difference in the levels of *RORα* transcripts in the blood lymphocytes between autistic and healthy children.

**Conclusion:**

The methylation of the *RORA* promoter region may not be considered as a common epigenetic risk factor for autism in all populations. Hence, the molecular pathogenesis of autism remains unclear in the population investigated.

## Introduction

Autism is a highly heterogeneous disease, to the extent that there are no two autistic children with 100% identity in features. Nevertheless, autism is characterized by four common features: impaired social communication and interaction, different language impairments, repetitive and stereotyped behaviors, often with restricted interests (1,2). Autism is the most devastating form of autism spectrum disorders (ASD) and the diagnostic criteria require that symptoms become apparent in early childhood, typically before age three. Autism has experienced an incredible increase in prevalence in the past three decades (3). Although genetic studies have suggested that schizophrenia and autism are highly heritable psychiatric disorders (4), they were, however, not successful in explaining the pathogenesis of autism. 

The role of epigenetic factors in the pathogenesis of autism was recently proposed and confirmed (5). For example, certain polymorphisms of the *MeCP2* gene, a global epigenetic regulator, were associated with an increased risk of autism (6). Methylation of the oxytocin receptor promoter have also been shown to be associated with autism (7). More recently, DNA methylation of 485,000 CpG sites in a pilot study of postmortem brain tissue (from three brain regions including dorsolateral prefrontal cortex, temporal cortex and cerebellum) from autistic cases were examined. This study revealed suggestive evidence for commonly altered methylation sites in ASD (8). 

The retinoic acid receptor-related orphan receptor alpha (*RORα*) also known as nuclear receptor subfamily 1, group F, member 1 (NR1F1) gene located on chromosome 15 (15q22.2), spans 741 kb and contains 11 exons. *RORA* encodes the transcription activator *RORα*, also known as NR1F1 with 556 aminoacids in length. *RORα* is a subfamily of the steroid hormone nuclear receptor superfamily and is involved in transcriptional regulation of many genes. Among its many known functions are regulation of the circadian clock, neuroprotection, survival and differentiation of Purkinje cells, and cerebellar development. All of these associated functions implicate *RORA* as a potential autism susceptibility gene with a significant role in autism pathogenesis (9). *RORα* is expressed in multiple tissues including testis, adipose tissue, kidney and liver. A high level of *RORα* expression has been observed in nerural tissues especially in the cerebellum and thalamus (10). The role of *RORα* in the process of brain development has not been widely studied beyond the cerebellum (11). However, for the first time, in 2010, Nguyen et al. (12) completed largescale DNA methylation profiling of lymphoblastoid cell lines derived from monozygotic twins discordant for autism and their nonautistic siblings. They observed decreased expression of *RORα* in the autistic brain and suggested *RORA* to be a novel candidate gene for autism pathogenesis. Moreover, *RORα*deficient mice showed various abnormalities including long thin bones (suggesting a role of *RORα* in bone formation), severe cerebellar atrophy and ataxia (13,14). It seems a possible link between *RORα* and pathogenesis of Spinocerebellar ataxia type 1 (*SCA1*) is via decreasing the number of cerebellar Purkinje cells (15). 

Given the role of *RORA* in the pathogenesis of nervous system disorders and its epigenetic impact on the incidence of autism, we aimed to investigate the methylation of the *RORA* promoter region in autistic cases. Moreover, considering the fact that other factors than methylation can affect gene expression, we also estimated the expression of *RORA* in autistic cases by real-time reverse transcriptase-polymerase chain reaction (RT-PCR). 

## Materials and Methods

### Selection of samples

This pilot experimental study was carried out after the approval of the Ethics Committee of the Behavioral Sciences Research Center of Isfahan University of Medical Sciences and Molecular and Cellular Research Center of Shahr-e Kord University of Medical Sciences. 

This study was performed on 30 autistic children (1 concordant monozygotic twin, 1 discordant monozygotic twin, two concordant dizygotic twins and 22 autistic individuals) who were under the care of Isfahan Society of Autistic Children. A relevant specialist had confirmed that they were autistic children. In the studied population, 24 cases were male and 6 cases were female. However, a total number of 10 normal siblings of the autistic cases (7 males and 3 females) were considered to be in-family control samples and 15 normal children from the urban regions of Isfahan were studied as population control samples after confirming their mental and physical health. All the studied cases were below 12 years of age. 

After completing the consent forms by parants, peripheral blood sample was collected from all cases. The methylation of a 136 bp fragment of a CpG island in *RORA* promoter region (including 13 CpG sites) was examined by melting curve analysis-methylation assay (MCA-Meth). 

### Isolation and bisulfite treatment of genomic DNA

Genomic DNA was isolated from lymphoblastoid cell lines (LCLs) using the QIAamp DNA Blood Mini-kit (Qiagen, Germany) and then underwent bisulfite treatment for methylation analyses. Two micrograms of genomic DNA was treated with sodium bisulfite using the Epitect Bisulfite Kit (Qiagen, Germany) according to the manufacturer’s instructions. 

After bisulfite treatment, DNA was amplified using specific primers that amplified 136 bp (64 to-200) of a CpG island in the promoter region of *RORA*. This primer set did not discriminate between methylated and unmethylated sequences. The primers and PCR conditions were specific for bisulfite-treated DNA, and did not amplify untreated DNA. For controls, the EpiTect Control DNA and Control DNA Set (Qiagen, Germany) were used as full methylated and full unmethylated DNA controls. 

Two primers: 

F: GTTAGYGTTGTTTGTGGTTAGA 

R: CCRACRAAAACAAAAAAAAAAAAACAAAC 

were designed for the region of interest using the Methprime (16) and Oligicalc online tools. 

All of the methylation analysis PCRs were performed using the Maxima SYBR Green/ROX Q-PCR Mastermix Kit (Thermoscientific) in a final volume of 20 μL, containing 1 μL bisulfite-treated DNA and a final concentration of 0.4 μmol/L for the forward and reverse primers. Reactions were incubated in a StepOne™ quantitative real-time PCR detection System (Applied Biosystems, USA) at 95˚C for 8 minutes, then 40 cycles of 95˚C for 50 seconds, 55˚C for 45 seconds and 72˚C for 45 seconds. Methylation was determined by the melting curve analysis of the PCR product at the end of the amplification cycle. The temperature was then ramped from 60 to 95˚C with increments of 0.3˚C at each step, waiting 15 seconds on the first step, then 5 seconds for each step thereafter. The derivative of fluorescence over temperature (dF/dT) was determined for each PCR product using the StepOne^tm^ Software v2.2 (Applied Biosystems, USA). The dF/dT curves of the samples were compared with those of the unmethylated and methylated controls. A sample was considered methylated when there was a shift in its dF/dT curve away from that of the unmethylated control. 

### RNA isolation and real-time polymerase chain reaction

The qRT-PCR analysis of *RORA* was performed to examine the correlation between the expression of the transcript and methylation status as predicted by MCA-Meth analysis. Total RNA was isolated using TRIzol reagents (Invitrogen, USA) following the manufacturer’s protocol. RNA was then reverse transcribed using RevertAid First Strand cDNA Synthesis Kit (Thermoscientific, USA) with oligo dT primers. 

The qRT-PCR was performed using SYBRGreen PCR Master mix with ROX (Fermentase, USA) in a final volume of 10 μL, containing 1 μL of cDNA and a final concentration of 0.4 μmol/L for forward and reverse primers. 

These primers: 

F: CAATGCCACCTACTCCTGTCC 

R: CTACGGCAAGGCATTTCTGTAAT 

were previously shown to successfully amplify *RORA* transcripts in skeletal muscle cells (17). Triplicate reactions were incubated in the StepOne™ quantitative real-time PCR detection system (Applied Biosystems, USA) at 95˚C for 10 minutes, then for 40 cycles of 95˚C for 15 seconds, 60˚C for 60 secf onds and 72˚C for 30 seconds. The gene of interest was then normalized against the reference gene glyceraldehyde-3-phosphate dehydrogenase (*GAPDH*). The relative expression of the target gene was calculated according to the 2^-ΔΔCt^ method as previously described (18). The PCR products were electrophoresed on 1.5% (w/v) agarose gels and stained with ethidium bromide to confirm expected product sizes. 

### Statistical analysis

Unpaired t test was used to statistically compare the mRNA expression level of *RORA*. A P value below 0.05 was considered significant. 

### Ethical considerations

All enrolled patients filled out the consent form to participate in this study according to the protocol of the Ethical Review Board of the Behavioral Sciences Research Center of Isfahan University of Medical Sciences. 

## Results

No methylation was observed in all studied CpG islands since the melting curve of all studied samples were comparable to that of the non-methylated control. 

**Fig.1:**
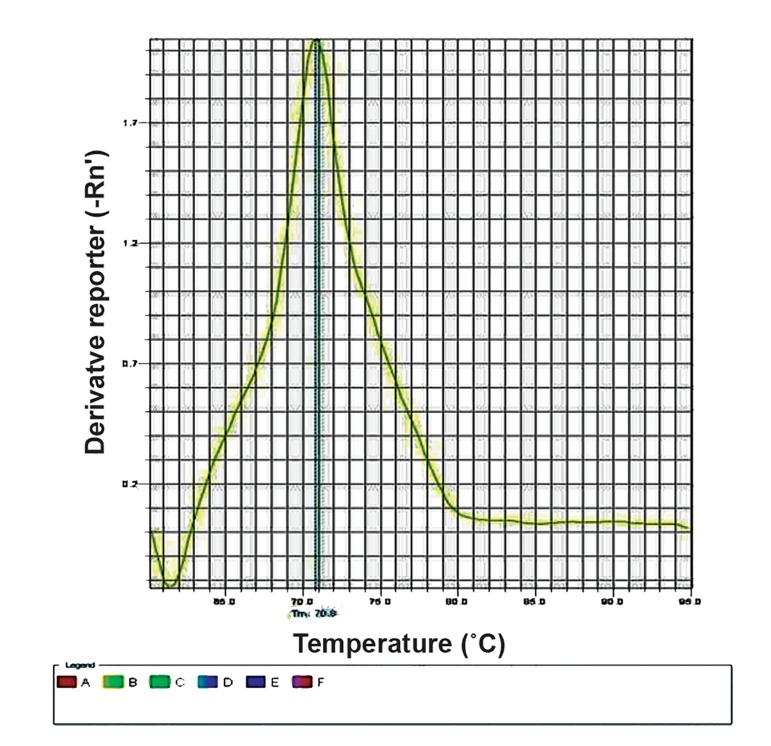
The melting curve of the non-methylated control.

**Fig.2:**
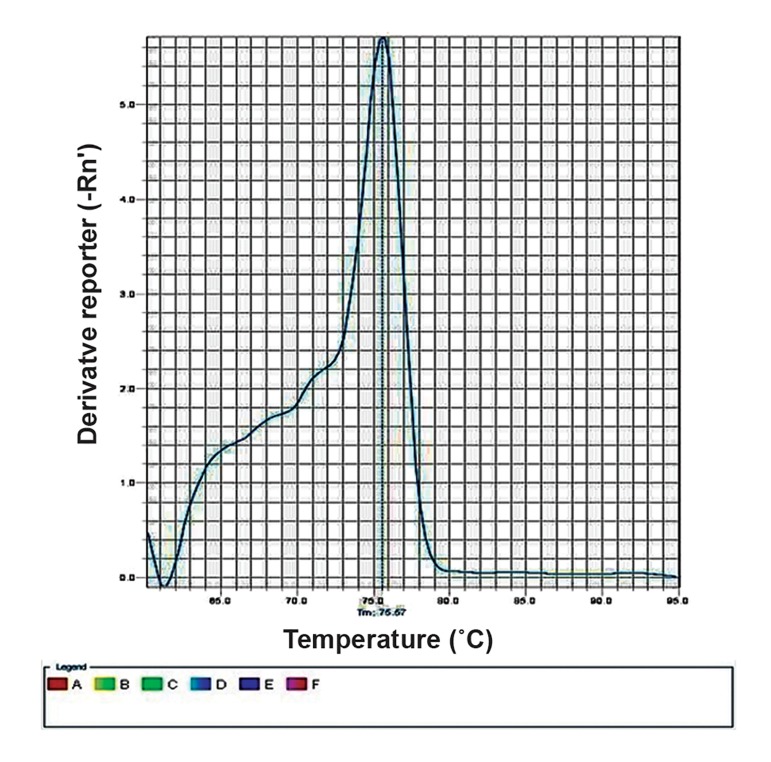
The melting curve of the methylated control.

**Fig.3:**
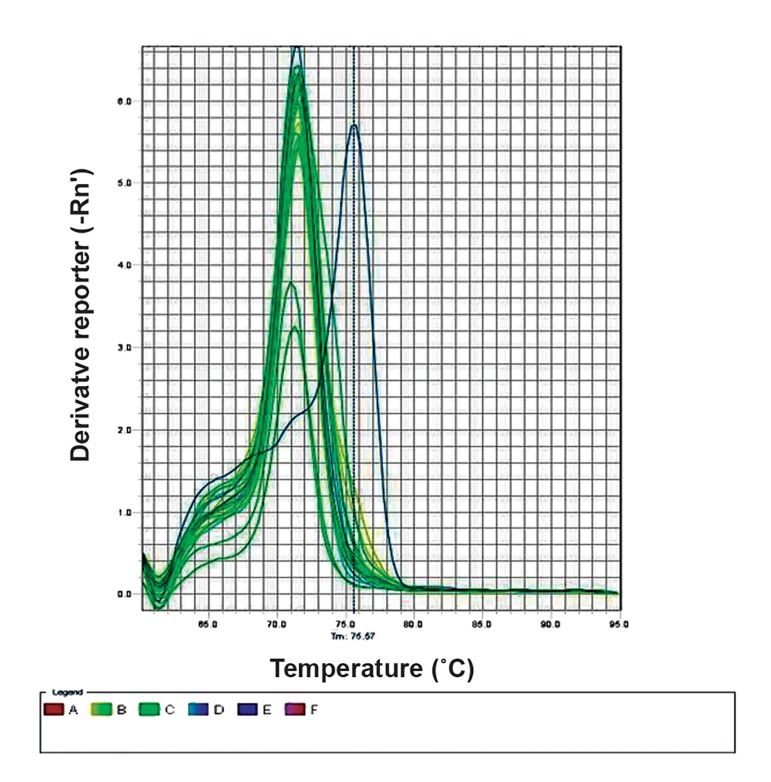
Sample melting curves show conformity with the nonmethylated controls.

The melting curve peak of the negative (completely non-methylated) and positive (completely methylated) controls was at 70.9˚C and 75.55˚C respectively (Fig.1, 2), showing a temperature range of 5˚C. The melting temperature (Tm) of the PCR products of all studied samples (including autistic cases, their normal siblings, and normal population controls) was 70.9˚C (Fig.3). This implies no methylation of any CpG site located in the promoter region of *RORA*. 

**Fig.4:**
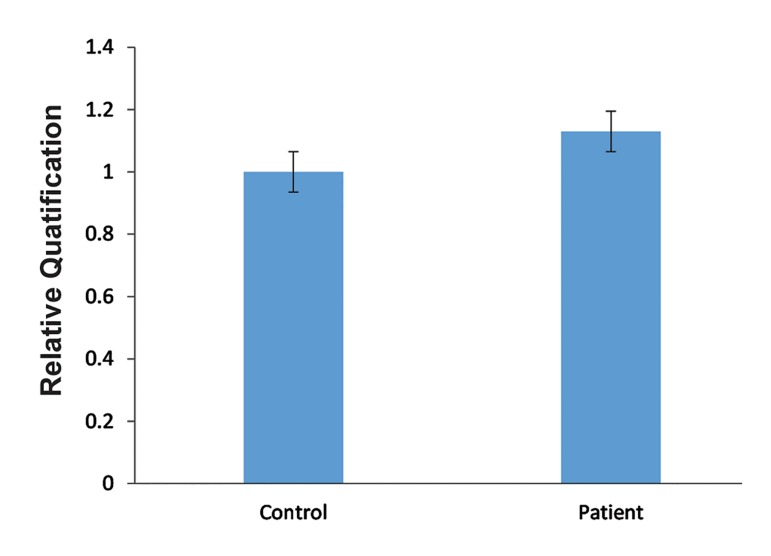
The comparison of *RORA* expression between controls and autistic samples (all data were normalized with *GAPDH* and compared with the control group expression.

The results of the qRT-PCR assay (Fig.4) indicated no significant difference in the levels of *RORA* transcripts in the blood lymphocytes between autistic and control samples. This finding was consistent with the results of the MCA-Meth analysis. 

## Discussion

This study applied the MCA-Meth assay on the lymphocyte cells of 30 autistic children in order to confirm the increased methylation of the promoter region of *RORA* gene and the consequent mRNA expression in autism pathogenesis in the Isfahan population. MCA-Meth protocol can detect the global methylation of a PCR-amplified region based on the difference between PCR melt curves and corresponding positive and negative control melt curves. Following bisulfate treatment, a methylated sequence of DNA will retain a higher GC/AT ratio which in turn will melt at a higher temperature compared with a non-methylated sequence (19). 

The melt curve of a sample will be considered to be positive for methylation if it shifts towards the positive control curve. MCA-Meth have some advantages over other DNA methylation screening methods specifically methylation-specific PCR (MSP) which include simplicity and no need for gel documentation, needing a little amount of genomic DNA (6-9 nanograms) compared with MSP (10-20 nanograms) (20), determination of the methylation quantity (in almost all CpG sites) and quality (methylated, non-methylated and semi-methylated conditions) in the considered sequence (in contrast with MSP), and the possibility of comparing it with bisulfate sequencing protocol in terms of specificity and sensitivity (21,22). 

*RORA* undertakes different tasks during the growth and development of human tissues including the nervous tissue, and similar to other genes it can be suppressed through the methylation of CpG islands (the most commonly known epigenetic mechanism) (23). 

The aim of this study is to assess the hypothesis (12) suggesting *RORA* silencing in autistic children by conducting a study on three pairs of discordant MZ twins for autism using microarray screening method and MSP confirmation. It should be noted that Nguyen et al. (12) also suggested a quantitative relationship between the severity of autistic phenotype and *RORA* gene expression. They also suggested that the expression level of *RORA* is regulated by DNA methylation on its promoter. However, our study did not confirm the above findings about the increased methylation of *RORA* promoter regions or the consequent decreased gene expression. 

Moreover, subsequent immunohistochemistry experiments showed a decreased protein level of this gene in the nervous tissue of post-mortem samples. They attributed the mRNA expression of *RORA* to its methylation because when global methylation was inhibited by 5-Aza-2deoxycytidine, the quantity of the mRNA transcription of *RORA* was increased by 1.8-fold only in the lymphocyte cells of autistic cases, but this did not happen either in their normal siblings or in normal twins. This implies that the decreased *RORA* expression may be attributable to the level of promoter methylation rather than any type of genetic mutation (12). 

The *RORα* as a nuclear receptor contributing to the transcription of a wide range of genes in humans. It therefore has a great potential in describing defective activities of brain in autistic children. Activities like circadian regulation by activating *BMAL1* (24), protecting brain against oxidative stress and inflammation (25), survival and differentiation of Purkinje cells (26,27) and cerebellar development (14) experience malfunction during autism. Collectively, *RORA* may be seen as a candidate gene for autism sensitivity. Moreover, *RORA* may shed light on an old question relating to an important hypothesis in autism, i.e. increased testosterone levels in the amniotic fluid of autistic children, reported about a decade ago (28). Sarachana et al. (29) agreed that, as a transcription factor, *RORα* can increase the aromatase enzyme level in the nervous system. This enzyme converts testosterone to estrogen in the nervous system. In fact, aromatase expression decrease as *RORA* expression decrease which, in turn, results in testosterone level increase in amniotic fluid and embryonic brain. 

Nevertheless, without ignoring the probable role of epigenome in the molecular pathogenesis of autism, our study could not confirm this association in autistic children of Isfahan. Surprisingly, Wong et al. (30) analyzed the methylome of 50 discordant autistic twins and although they identified a considerable number of methylated genes, *RORA* showed no significant difference between autistic and normal children. 

The following reasons may clarify this inconsistency. The first is that the promoter methylation of *RORA* and the consequent decreased mRNA expression in the nervous tissues may not occur in lymphocyte cells. It is accepted that DNA methylation can differ cell by cell. On this basis, this methylation mosaicism can be expected in different tissues, especially in the event of hypermethylation (like *RORA* promoter methylation), during cell growth and differentiation. However, there is no detailed information about its probable mechanisms. The second reason pertains to the technical differences of the studies. Although MSP is a simple, fast method for screening promoter methylation, it is, however, limited to testing the methylation status in just one CpG site. Therefore, compared with MCA-Meth, it shows a weak performance in detecting the methylation of a genomic region. Our study considered 13 CpG sites in the promoter region of *RORA* and confirmed no DNA methylation in the target sites. Some studies have compared the capability of MSP and MCA-Meth in detecting a methylated region and reported that MCA-Meth has a higher capability (21). Therefore, it can be argued that although both techniques are valuable, they may report different results for the methylation of a target region. 

The third possibility is that the methylation reported by Nguyen et al. (12) in the American population may be associated with an environmental factor not experienced by the population studied here. In other words, since the tissue mosaicism of DNA methylation is an accepted phenomenon, it is probable that this epimutation has an environmental clue in different populations. 

The results of our study may disagree with those of Nguyen et al. (12) study from a genomic point of view because it has not yet been discovered which CpG site in the *RORA* promoter accompanies the gene silencing. It is therefore probable that the methylation of an CpG nucleotide outside of the considered region (but inside the *RORA* promoter region) may be associated with its decreased expression. For example, a number of studies on *TIMP3* gene have emphasized that only one region of the three overlapping regions of the *TIMP3* promoter CpG island has a relationship with its silencing. On this basis, it can be argued that, at least in some genes, the methylation of specific CpG sites in the CpG island plays a greater role in gene silencing than other sites. Therefore, the *RORA* promoter CpG island may follow such epigenetic pattern. 

## Conclusion

Nevertheless the methylation of *RORα* promoter region may not be considered as a common epigenetic risk factor for autism in all populations. Hence, the molecular pathogenesis of autism remains unclear in the population investigated. 
